# Spontaneous hemothorax caused by ruptured multiple mycotic aortic aneurysms: a case report and literature review

**DOI:** 10.1186/s13019-017-0665-6

**Published:** 2017-12-02

**Authors:** Po-Sung Li, Chung-Lin Tsai, Sung-Yuan Hu, Tzu-Chieh Lin, Yao-Tien Chang

**Affiliations:** 1 Department of Emergency Medicine, Taichung Veterans General Hospital, Taichung, Taiwan; 2 Division of Cardiac Surgery, Cardiovascular Center, Taichung Veterans General Hospital, Taichung, Taiwan; 3 School of Medicine, Chung Shan Medical University, Taichung, Taiwan; 4 Institute of Medicine, Chung Shan Medical University, Taichung, Taiwan; 5 Department of Nursing, College of Health, National Taichung University of Science and Technology, Taichung, Taiwan; 6 Department of Nursing, Central Taiwan Univeristy of Science and Technology, Taichung, Taiwan; 7 College of Public Health, China Medical University, Taichung, Taiwan; 8 1650 Taiwan Boulevard Sect. 4, Taichung, 40705 Taiwan

**Keywords:** Computed tomography (CT), Hemothorax, Mycotic aortic aneurysm (MAA)

## Abstract

**Background:**

Mycotic aortic aneurysm (MAA) is a rare clinical entity with an incidence of 1-3%, but it is a life-threatening infection of aorta characterized by dilatation of aorta with false lumen. Multiple MAAs have been reported rarely with an incidence of 0.03% and associated with a high mortality rate of 80% if ruptured.

**Case presentation:**

A hypertensive and diabetic 78-year-old man visited our emergency department complaining intermittent dull and tingled pain over the left flank region for 1 week. Chest X-ray showed left pleural effusion and hemothorax was confirmed by thoracocentesis. Computed tomography (CT) of chest demonstrated multiple thoracic aortic aneurysms and the pathological findings disclosed the diagnosis of multiple MAAs. He was discharged under an uneventful condition post-surgical aortic repair with adequate intravenous antibiotics for 4 weeks.

**Conclusions:**

CT scan may make a definite diagnosis of multiple MAAs and management with surgical debridement, aortic repair and full-course antibiotics for Gram-positive coccus and/or Gram-negative bacillus is recommended.

## Background

Hemothorax is the presence of blood in the pleural space. The source of blood may be trauma to the chest wall, lung parenchyma, heart, or great vessels, infection/ inflammation, malignancy, coagulopathy or congenital arteriovenous malformations. Mycotic aortic aneurysm (MAA) is a rare clinical entity with an incidence of 1-3%, but it is a life-threatening infection of aorta characterized by dilatation of aorta with false lumen [1–5]. Multiple MAAs have been reported an incidence of 0.03% and associated with a high mortality rate of 70-80% if ruptured. Spontaneous hemothorax associated with ruptured MAAs has been reported rarely [6].

## Case presentation

**Fig. 1:**
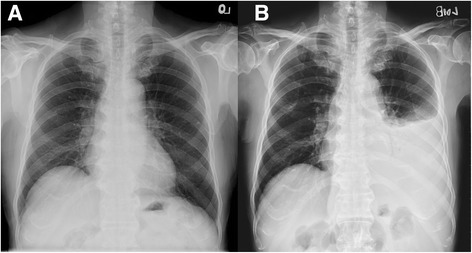
Chest X-rays showed normal heart size, sharp cardiopleural angle, bare stent of coronary artery and no significant of aortic lesion (Panel **a**). Chest X-rays revealed left pleural effusion and widening of upper descending aorta (Panel **b**)

**Fig. 2:**
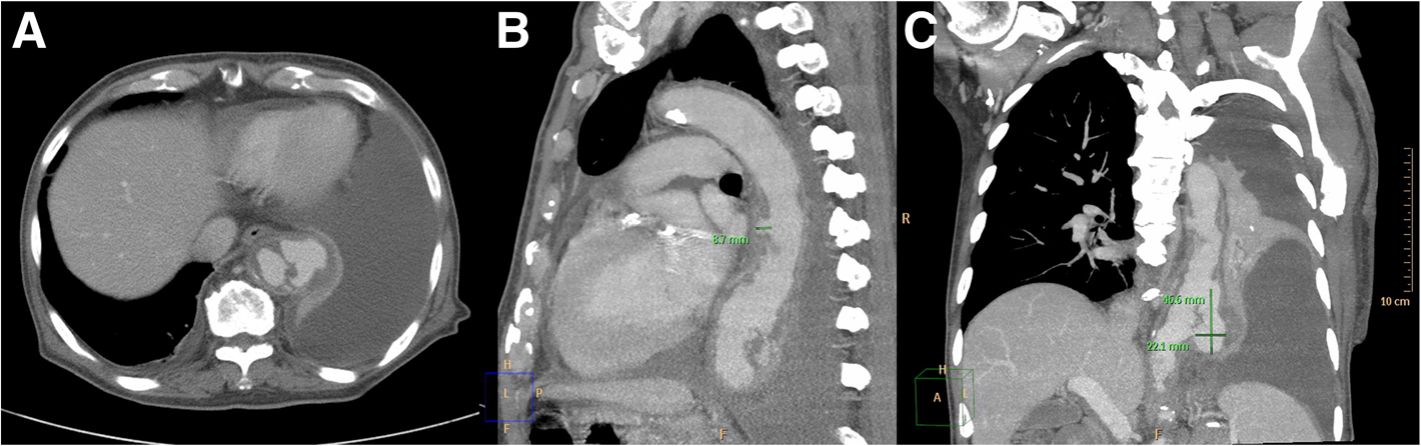
Axial, sagittal and coronal views of computed tomographic scan with intravenous contrast media of chest showed irregular dumbbell-shaped aortic lesion (Panel **a**), multiple mushroom-like thoracic aortic aneurysms with the size of 8.7 mm in diameter (Panel **b**) and 46.6 mm × 22.1 mm (Panel **c**) with periaortic soft tissue density at the level of between 8th and 11th thoracic spine, massive pleural effusion and atelectasis of left lung

**Fig. 3:**
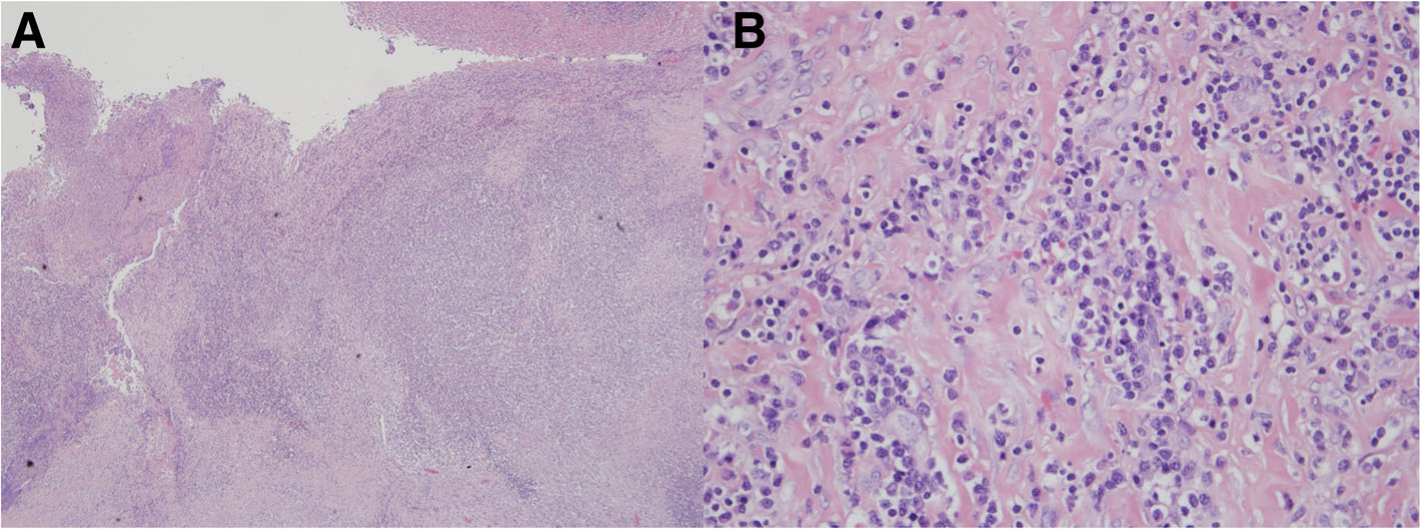
Pathological findings disclosed fibrinous material coating on the internal luminal surface and formation of focal abscess was noted in the destructive vascular wall (×100) (Panel **a**). There were infiltrations of acute and chronic inflammatory cells in the damaged vascular wall (×1000) (Panel **b**)

A 78-year-old hypertensive and diabetic man had a history of coronary artery disease with percutaneous coronary intervention in 1997. Chest X-ray (Fig. 1a) was normal 11 months ago. He denied major chest trauma recently. He suffered from intermittent low grade fever, dull and tingled pain over left flank region and progressive dyspnea for 1 week. He was brought to our emergency department. On arrival, vital signs were a respiratory rate of 22 breaths per min, a heart rate of 125 beats per min, a blood pressure of 158/79 mmHg and a body temperature of 36.6 °C. Physical examination revealed a pale conjunctiva, no heart murmur, absent breathing sound of left lower lung, and knocking pain over left costovertebral angle. Significant laboratory evaluation revealed white blood cell counts (WBCs) of 13,700/mm^3^ with 76.3% of segmented neutrophils, hemoglobin of 7.8 g/dl, red blood cell counts (RBCs) of 4.26 × 10^6^/mm^3^, platelet counts of 634 × 10^3^/mm^3^, creatinine 1.5 mg/dl, albumin 2.6 g/dl, protein 7.8 g/dl, lactate dehydrogenase (LDH) 384 U/l, glucose of 257 mg/dl, and high-sensitivity C-reactive protein (hs-CRP) of 18.6 mg/dl. Chest X-ray showed left pleural effusion and mild widening of upper descending aorta (Fig. 1b). Blood-tinged pleural effusion was confirmed via a fine-needle aspiration under sono-guide after informed consent of patient. Analysis of pleural effusion were exudative and infection of pleural effusion should be considered, including WBCs of 1625/mm^3^, with neutrophils of 62% and lymphocytes of 31%, RBCs of 12,500/mm^3^, protein of 4.5 g/dl, LDH of 266 U/l, glucose of 201 mg/dl and specific gravity of 1.030. Fever, flank pain, leukocytosis, elevated hs-CRP and hemothorax were clinical clues for high suspicion of ruptured infectious aorta. Computed tomography (CT) with intravenous contrast media depicted ruptured multiple thoracic aortic aneurysms at the level of between 8th and 11th thoracic spines with atelectasis of left lung and massive hemothorax (Fig. 2), so emergency surgical intervention with resection of fragile aorta, debridement of involved periaortic soft tissue, and reconstruction for descending aorta with Dacron graft of 20 mm was performed. Although the cultures of blood, pleural fluid, and resected aortic tissue showed no growth of bacteria or mycobacterium tuberculosis, the pathological findings demonstrated injury of vascular wall with acute and chronic inflammatory cells infiltration, fibrinous material coated on the internal luminal surface, focal abscess formation in vascular wall (Fig. 3) and no malignancy cells or granulation tissue. The serological screen for syphilis was non-reactive. The patient recovered gradually after surgical aortic repair, intensive care and adequate intravenous antibiotics for cover Gram-positive and Gram-negative bacteria with prostaphlin 2 g per 6 h and ceftriaxone 2 g per day for 4 weeks despite of negative-culture result. He was discharged under an uneventful condition with a regular follow-up of cardiovascular out-patient department for 2 years.

## Discussion

Mycotic aortic aneurysm (MAA) is a rare but life-threatening infection with an incidence of 1~3% [1–5], characterized either by bacteremic seeding from extracardiac septicemia with bacterial invasion of an atherosclerotic vascular wall, endocarditis and contiguous/direct infection, leading to weakening and dilatation of vascular wall with formation of false lumen [5–7]. Multiple MAAs had been reported an incidence of 0.03%, which had a high risk of rupture with a mortality rate of 80%. Hemothorax as a sign of ruptured multiple MAAs was extremely rare and only few cases had been reported previously [6].

The differential diagnosis of hemothorax implicated the possibility of life-threatening underlying illnesses such as trauma, coagulopathy, spontaneous pneumothorax, vascular lesions, spontaneous esophageal rupture, infectious disease (such as pneumonia, tuberculous pleurisy, fungal disease, subphrenic abscess), malignancy, connective tissue disease (lupus pleuritis, rheumatoid pleurisy, Wegener’s granulomatosis), pulmonary embolism, pulmonary sequestration, and endometriosis. They are difficulty to be differentiated from chest radiographs or clinical presentations. The rupture of thoracic mycotic aortic aneurysm (MAA) should be considered in the differential diagnosis of hemothorax [1–7].

MAA can develop either by infection of arterial wall or previous aneurysm with secondary infection. They maybe have led to a true or false lumen. Blood cultures are positive in 50-75% of MAA patients [2, 5, 8]. MAA is diagnosed according to clinical presentations with triad of fever, pain and pulsatile mass; laboratory investigations of leukocytosis and elevation of inflammatory biomarkers; radiological typical findings, including mushroom-like appearance, new aneurysm formation, rapid expansion or morphological change of known aneurysms, synchronous lesions, intramural or perivascular gas, edema, soft tissue mass or stranding, ring enhancement, disruption or disappearance of aortic calcification in late stage, and extravasation in rupture; cultures of blood and/or resected aortic tissue; and the histopathological features of an acute infection, including abscess and infiltration of neutrophils [2, 5, 7].

Most common pathogens are bacteria such as *Staphylococcus* spp., *Salmonella* spp*.*, and *Treponema pallidum*. The risk factor includes atherosclerosis, male sex, cigarette smoking, vascular abnormalities (pre-existing aneurysms), arterial trauma, old age, immunocompromised status, intravenous drug abusers, and infectious endocarditis. Medical treatment alone can’t complete cure, so cardiovascular surgeons must perform surgical debridement and reconstruction of vascular continuity for thoracic aortic multiple MAAs plus intravenous antibiotics of 4-6 weeks at least for Gram-positive coccus and Gram-negative bacillus [2, 5, 9–12].

The natural history of untreated mycotic aneurysms is of fatality from either massive hemorrhage or fulminant sepsis. Complications include 45% of rupture and 18% of fistula formation in late stage [5]. If an aortic mycotic aneurysm is diagnosed, surgical removal of an infected aneurysm must always be required because of a high mortality rate of 70-80% due to a high risk of rupture of mycotic aneurysm. Therefore undue delay of surgical intervention should be avoided. Emergency operation is recommended in those who have a ruptured aneurysm or are septic and unstable [6, 13]. The optimal timing and best surgical procedure of surgical intervention are still difficult to be determined. The timing of surgical intervention should be determined by estimated risk of aneurysm rupture and surgical risk according to the patient’s underlying condition and short-interval CT re-examinations [13, 14]. The challenges of surgical intervention in patients with mycotic aneurysms are re-infection, difficulties of anastomosis lines in fragile cut-end tissue, early and late postoperative bleeding, so a wide and extensive debridement of all infected tissue and a resection back to the healthy and non-infected wall of the aorta are mandatory [15, 16]. In our case, he received emergency surgical repair immediately due to ruptured multiple MAAs after radiological and laboratory survey. He was fully recovered and received regular follow up at our hospital for 2 years.

## Conclusion

Although the incidence is rare, the cause of spontaneous hemothorax should include rupture of MAA. Hold the implantation of chest tube if there is no trauma-related hemothorax. Etiologies of spontaneous hemothorax must be completed as soon as possible. Emergency surgical repair with adequate and full course of intravenous antibiotics are recommended if there is an evidence of rupture of MAA, such as hemothorax, because of high mortality. We recommend that the differential diagnosis of spontaneous hemothorax in high suspicion of infectious aorta should include MAA.

## Abbreviations

CT: Computed tomography
LDH: Lactate dehydrogenase
MAAs: Mycotic aortic aneurysms
RBCs: Red blood cell counts
WBCs: White blood cell counts
